# Cardiovascular haemodynamics in pre-eclampsia using brain naturetic peptide and tissue Doppler studies

**DOI:** 10.5830/CVJA-2013-023

**Published:** 2013-06

**Authors:** DP Naidoo, S Fayers, J Moodley

**Affiliations:** Department of Cardiology, University of KwaZulu-Natal, Durban, South Africa; Department of Obstetrics and Gynaecology, University of KwaZulu-Natal, Durban, South Africa; Womens’ Health and HIV Research Group, University of KwaZulu-Natal, Durban, South Africa

**Keywords:** pre-eclampsia, brain natriuretic peptide, tissue Doppler, echocardiography

## Abstract

**Aim:**

To determine early haemodynamic changes in pre-eclampsia (PE) using tissue Doppler echocardiography and brain natriuretic peptide (BNP), and to relate these changes to obstetric outcomes.

**Methods:**

Consenting primigravidae patients in the third trimester of pregnancy were included in the study, which was carried out in a large regional hospital in Durban, South Africa; 115 primigravidae (52 pre-eclamptics and 63 normotensive pregnant patients) attending the maternity unit including the antenatal clinics at the study site were studied. The patients, matched for maternal and gestational age, were examined during pregnancy and within the puerperium. Transthoracic echocardiography (TTE), tissue Doppler imaging (TDI), umbilical artery Doppler and laboratory investigations were performed.

**Results:**

BNP levels were significantly increased in the antepartum period [23.8 (2–184.1) vs 6.0 (0.5–45.2) pmol/l; *p* < 0.0001] and during labour [15.0 (1.8–206.4) vs 8.7 (1.9–24.8) pmol/l; *p* < 0.01] in the pre-eclamptic group compared to the normotensive controls. In the postpartum period, mean BNP levels were 4.2 (1.7–51.4) and 5.95 (2.2–38.7) pmol/l in the pre-eclamptic and normotensive groups, respectively (*p* > 0.05). Tissue Doppler Em/Ea ratios were elevated in the pre-eclamptic compared to the normotensive group (11.02 ± 5.6 vs 9.16 ± 2.6; *p* < 0.05). Mean left atrial size was larger (38 mm) in the pre-eclamptic group than in the normotensive group (35 mm) but this difference was not significant (*p* > 0.05).

The umbilical artery resistance index was significantly higher in the pre-eclamptic group compared to the normotensive group (0.68 ± 0.06 vs 0.63 ± 0.05; *p* < 0.001). There was an increased rate of Caesarean sections performed in the pre-eclamptic group (*n* = 24) compared to the normotensive group (*n* = 18; *p* < 0.001). There were two stillbirths in the pre-eclamptic group and none in the normotensive group. As expected, birth weight (2.6 ± 0.8 kg vs 3.14 ± 0.42 kg; *p* < 0.0001) was lower in the pre-eclamptic group compared to the normotensive group.

**Conclusion:**

In pregnancies complicated by pre-eclampsia, BNP levels were increased in comparison to normotensive pregnancies and this was accompanied by early changes in left ventricular diastolic function as determined by the tissue Doppler E_m_/E_a_ ratios. These changes reverted to baseline values, as indicated by return of BNP levels in the pre-eclamptic group to levels seen in the normotensive group. These changes were associated with an increased Caesarean section rate and lower birth weights in pre-eclamptic mothers.

## Abstract

Hypertensive disorders of pregnancy complicate approximately 10–16% of pregnancies and constitute one of the leading causes of maternal, foetal and neonatal morbidity and mortality worldwide,^1^,^2^ particularly in low- and middle-income countries.^3^ Most, if not all of the morbidity and mortality in hypertensive disorders of pregnancy arise from pre-eclampia (PE) and its complications.^4^

Pre-eclampsia arises when expression of pro-inflammatory, anti-angiogenic and angiogenic factors lead to a systemic endothelial cell dysfunction with exaggerated inflammatory and vasoconstrictor responses.^5^,^6^ Vasoactive hormones play an important role in the pathogenesis of PE, linking placental hypoperfusion with hypertension, systemic disease and proteinuria.

Until recently most of the non-invasive studies of the haemodynamic changes of PE have employed two-dimensional echocardiography to measure cardiac dimensions and systolic function. Until the advent of tissue Doppler imaging (TDI), there have been few sensitive echocardiographic measures of changes in cardiac filling pressure.

Since vasoconstriction and volume retention characterise PE, it is possible that TDI studies and biomarkers of cardiac stress may be useful in detecting early haemodynamic changes in PE. To date, several different biophysical and biochemical markers have been investigated. These include sflt-1, VEGF, soluble endoglin, P-selectin and PP-13, which reflect the pathogenesis of PE,^7^,^8^ and more recently, haemodynamic changes have been documented with brain (or B-type) natriuretic peptide (BNP).^9^

Measurement of both BNP and NT-proBNP have been shown to be sensitive markers for the detection of mild systolic or diastolic heart failure or asymptomatic left ventricular dysfunction,^10^,^11^ and for the diagnosis of congestive heart failure in patients with dyspnoea in an acute-care setting.^12^ Recent studies suggest that BNP levels are elevated in pre-eclamptics.^13^-^16^

We hypothesised that there may be alterations in the BNP levels associated with pre-eclampsia in response to changes in cardiac myocyte stretch and that this may may reflect pressure/volume changes in the cardiac ventricles in this unique disorder of pregnancy. There are no studies that correlate BNP values with structural and/or functional changes in ventricular function in PE during late pregnancy and the postpartum period. Our objective was to characterise the serial changes in the BNP levels in the third trimester of pregnancy and the early postpartum period. We performed Doppler studies of the heart and umbilical cord, measured plasma BNP levels and evaluated obstetric outcomes in PE and normotensive healthy pregnant patients.

## Methods

Primigravidae without any pre-existing history or clinical evidence of hypertension/cardiac or renal disease were included in the study after having obtained informed consent. PE was diagnosed when women in their first pregnancy had a blood pressure level of at least 140 mmHg systolic and 90 mmHg diastolic pressure on two occasions over four hours apart for the first time after the 20th week of pregnancy, associated with at least one plus of proteinuria on urinary dipstick. For each patient with PE, at least one healthy normotensive patient was enrolled in the study. At the time of enrolment the study participants were not on any medications to lower blood pressure.

A full history and clinical examination was performed. Systolic and diastolic blood pressures were recorded by automated readings (Dinamap) after a 30-min period of rest in the sitting position. Baseline blood investigations included a full blood count, urea and creatinine, urates and BNP levels. Obstetric ultrasound examination was performed on all patients. Foetal wellbeing was assessed sonographically and by Doppler umbilical flow measurements in relation to appropriate growth for gestational age, amniotic flow index and placental sufficiency.

Pregnancies were followed and timing and mode of delivery were noted. Apgar scores and birth weight were recorded and any admissions to the neonatal intensive care noted. All babies were asseded at birth and seven days after delivery. A total of 63 normotensive pregnant women with similar age and ethnicity without a history of cardiovascular disease, pulmonary or systemic hypertension served as controls.

In this study, blood for BNP estimations from study participants were obtained at three pre-specified time points, i.e. at the time of recruitment (28–40 weeks), intra-partum, and the last specimen was collected between day one and seven post delivery. The samples were collected in plastic specimen tubes containing ethylenediamine tetra-acetic acid (EDTA) and transported on ice to the laboratory where they were centrifuged.

The plasma was stored at −20°C and NT-proBNP was assayed in batches by standard electrochemiluminescence immunoassay (ECLIA) using the Modular Analytics E170 (ELECYS module) and Elecsys 1010/2010 analyzer (Roche diagnostics,). According to the National Committee for Clinical Laboratory Satndards (NCCLS), the resting BNP values considered normal for this methodology lie below 100 pg/ml. The within-assay and total precision coefficients of variation for NT-proBNP mean 208 pmol/l is 0.8 and 4.5%, respectively. The reading sensitivity is < 2.0–5 000 pg/ml (0.58–1 445 pmol/l).

## Echocardiography and TDI

Shortly after enrolment standard two-dimensional directed M-mode Doppler echocardiography followed by TDI was performed with the patient in the left decubitus position. Doppler echocardiography was performed using a HDE 11 imaging system (Philips) with a phased-array transducer and an emission frequency of 3.0 MHz.

The left ventricular (LV) end-systolic and end-diastolic dimensions, LV wall thickness, and left atrial (LA) dimensions were measured according to the American Society of Echocardiography guidelines using the leading edge method.^17^ The left atrial volume was estimated using the biplane ellipsoid formula. The LV end-systolic and end-diastolic volumes and the ejection fraction were measured from the apical four-chamber view using the modified Simpson’s method. TDI was performed with transducer frequencies of 1.8–3.6 MHz with as minimum optimal gain as possible to obtain the best signal-to-noise ratio.^18^

## Foetal ultrasound and umbilical artery Doppler

Foetal biometrical ultrasound was performed using a Toshiba (Nemio) scanner in B-mode and a low-frequency (3.75 MHz) curvilinear probe.^19^ Umbilical artery Doppler studies were then performed using pulsed-wave Doppler to measure flow velocity and calculate the resistance index (RI) as follows: peak systolic velocity was divided by the sum of measurements at peak systole and diastole [RI = systole/(systole + diastole)] and averaged over three cardiac cycles.

## Statistical analysis

SPSS version 11.5 (SPSS Inc. Chicago Ill, USA) was used for statistical analysis. The baseline characteristics were reported as mean ± standard deviation and were compared between the two groups using the Fisher exact test for categorical variables and the Student’s *t*-test for continuous variables. Outcome measures were BNP levels, echocardiographic and TDI findings and obstetric outcomes in both groups.

Chi-square statistics or Fisher’s exact tests were used where appropriate to examine associations between categorical exposures and outcomes. Independent two-sample *t*-tests were used to compare mean BNP levels between two categories. Results are presented as mean and range in brackets. As BNP levels were not normally distributed, these are presented as median values and a Mann-Whitney *U*-test was used to compare the two groups. Spearman’s test was used for correlation studies. A *p*-value of < 0.05 was considered as statistically significant.

Ethics approval was obtained from The University of KwaZulu-Natal Biomedical Research Ethics Committee.

## Results

One hundred and fifteen primiparous patients (63 normotensive and 52 pre-eclamptics) were recruited (Table 1). A total of 113 participants had complete longitudinal BNP values for all three pre-specified time periods. There was no difference in gestational age at entry and in mode of delivery in both groups.

**Table 1:** Demographic Data Between Groups

*Parameter*	*Normotensive (n = 63)*	*Pre-eclamptic (n = 52)*	p*-value*
Age (years)	20.4 ± 3.7	21.5 ± 4.7	< 0.06
BMI (kg/m^2^)	27.9 ± 5.5	29.4 ± 7.9	< 0.05
Gestational age at entry (weeks)	34.5 ± 2.7	34.3 ± 2.7	ns
Gestational age at delivery (weeks)	38.4 ± 1.9	37.8 ± 2.2	ns
Urine dipstick	0 (0–1+)	2+ (1+ –3+)	< 0.0001
Oedema	0 (0–1+)	2+ (1+ –4+)	< 0.0001
HIV status positive	17	17	
CD_4_ count < 200 (cells/mm)	5	5	
SBP (mmHg)	127.03 ± 12.9	163.2 ± 17.6	< 0.0001
DBP (mmHg)	78.32 ± 8.6	104.4 ± 11.9	< 0.0001
Pulse (bpm)	84.7 (72–96)	86.0 (72–106)	< 0.04

Values are expressed as mean ± standard deviation. Actual counts are reported for HIV status and CD4 count. SBP = systolic blood pressure; DBP = diastolic blood pressure; bpm = beats per minute.

The mean age of the pre-eclamptics was slightly higher (21.5 ± 4.7 vs 20.4 ± 3.7 years) but this difference was not significant (*p* < 0.06). The body mass index in the pre-eclamptic group was significantly higher compared to the normotensive pregnancies (29.4 ± 7.9 vs 27.9 ± 5.5; *p* < 0.05). There were 17 patients in each group who were HIV infected, five of whom in each group had CD_4_ cell counts < 200 cells/mm.

Most patients were managed with the following antihypertensive agents: methyldopa or nifedipine XL orally. Where very high blood pressure values were not adequately controlled, combination antihypertensive therapy using intravenous hydralazine or labetolol with the addition of magnesium sulphate was used.

Laboratory analysis (Table 2) showed lower platelet counts in the pre-eclamptic group; 10 patients in the pre-eclamptic group developed thrombocytopaenia and one in the normotensive pregnancies. As expected, serum creatinine, urea, uric acid and urine protein levels were significantly elevated in the pre-eclamptic group. There were no differences in gestational age and estimated foetal weight between groups. Polyhydraminos was present in one normotensive patient and in six pre-eclamptic patients. The umbilical artery resistance index was significantly higher in the pre-eclamptic group compared to the normotensive group (0.68 ± 0.06 vs 0.63 ± 0.05; *p* < 0.0001).

**Table 2:** Baseline Characteristics

*Parameter*	*Normotensive (n = 63)*	*Pre-eclamptic (n = 52)*	p*-value*
Full blood count			
Hemoglobin (g/dl)	10.9 ± 1.3	11.3 ± 1.4	ns
Platelets (× 10^9^/l)	251.8 ± 82.8 33.1 ± 3.1	215.5 ± 82.6	< 0.02
Haematocrit (l/l)		34.2 ± 3.6	< 0.07
Blood chemistry	2.5 ± 0. 7		
Urea (mmol/l)	54.5 ± 8.9	3.08 ± 1.18	< 0.002
Creatinine (μmol/l)	0.27 ± 0.06	62.4 ± 12.7	< 0.0001
Uric acid (mmol/l)		0.33 ± 0.07	< 0.0001
Urine dipstick	0 (0–1+)		
Proteinuria		2+ (1+ –3+)	< 0.0001
Resistance index	0.63 (0.5–0.62)	0.68 (0.62–0.84)	< 0.0001
Gestational age	34 (28–39)	34 (26–39)	ns
Estimated foetal weight	2.6 (1.2–3.7)	2.5 (0.8–3.8)	ns
Adequate liquor	60	42	ns

Echocardiographic and BNP findings are shown in Table 3. There were no differences in the left ventricular chamber dimensions and ejection fraction between the two groups. The left atrium diameter was slightly increased in the pre-eclamptic group but this difference was not significant. Estimates of the left ventricular filling pressure as measured by tissue Doppler E/E_a_ ratio were significantly higher in the pre-eclamptic group compared to the normotensive pregnancies (11.02 ± 5.6 vs 9.16 ± 2.6; *p* < 0.05).

**Table 3:** Echocardiographic And BNP Findings Within Groups

*Parameter*	*Normal range*	*Normotensive (n = 41)*	*Pre-eclamptic (n = 36)*	p*-value*
LV diastole (mm)	35–55	55 49 (37–56)	50 (39–60)	ns
LV systole (mm)	23–34	33 (23–40)	34 (24–41)	ns
Fractional shortening (%)	27–35	32 (27–42)	32 (26–38)	ns
Ejection fraction (%)	30–60	64 (54–62)	64 (56–66)	ns
LV posterior wall (mm)	6–10	7 (5–9)	7 (5–10)	ns
Septal thickness (mm)	6–10	7 (5–9)	7 (4–10)	ns
Left atrium (mm)	19–39	35 (25–41)	38 (32–55)	ns
Aortic root (mm)	20–37	23 (21–29)	24 (19–30)	ns
Tissue Doppler (E_a_)	< 8	9.2 (4.5–12.25)	11.0 (7.3–15.4)	< 0.05
BNP (median) (pg/mol)				
Antepartum (1)	6.0 (0.5–45.2)		23.8 (2–184.1)	< 0.0001
Labour (2)	8.7 (1.9–24.8)		15.0 (1.8–206.4)	< 0.01
Postpartum (3)	5.95 (2.2–38.7)		4.2 (1.7–51.4)	< 0.01

Median values for all parameters with range in brackets

**Fig. 1.**
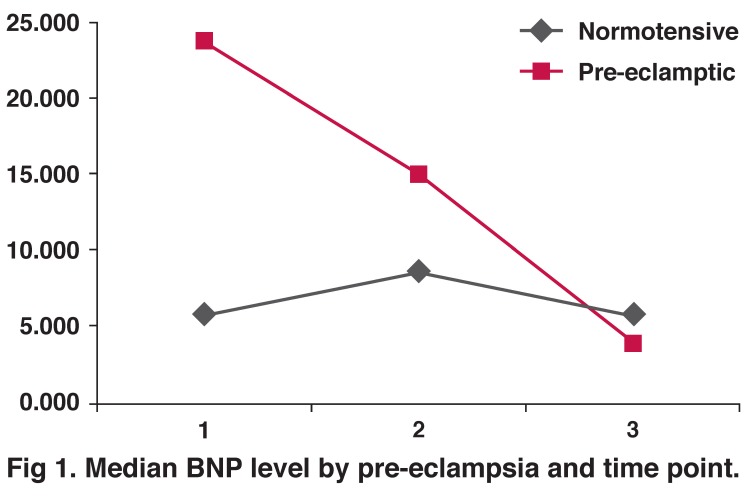
Median BNP level by pre-eclampsia and time point.

There were significant differences between the NT-proBNP levels in all pre-specified time points between the PE and normotensive controls except post delivery (Fig. 1). NT-proBNP levels were significantly increased in the antepartum period in the pre-eclamptic group compared to the normotensive group [(2–184.1) vs 6.0 (0.5–45.2) pmol/l; *p* < 0.0001]. BNP levels rose during labour (1.8–206.4 vs 1.9–24.8 pmol/l; *p* < 0.01) and subsided in the postpartum period (1.7–51.4 vs 2.2–38.7 pmol/l; *p* < 0.01). There was a weak positive correlation between baseline NT-proBNP and TD (*r* = 0.22; *p* < 0.051), and a stronger correlation between baseline NT-proBNP and the resistance index (*r* = 0.321; *p* < 0.001).

The number of maternal complications was higher in the pre-eclamptic group compared to the normotensive group (Table 4). In the normotensive group, 38 patients delivered vaginally and 18 were delivered by Caesarean section (CS), while in the pre-eclamptic group, 24 were delivered by CS and 27 delivered by the vaginal route. The CS rate was significantly higher in the pre-eclamptic group (24 vs 18; *p* < 0.05).

**Table 4:** Maternal And Foetal Outcome

*Parameter*	*Normotensive pregnancies (n = 62)*	*Pre-eclamptic (n = 48)*
Thrombocytopaenia	1	10
Pulmonary oedema	0	2
Eclampsia	0	11
HELLP syndrome	0	1
Birth weight mean ± SD (kg)	2.64 ± 0.8	3.14 ± 0.42
Apgar scores		
1 min	7 (0–0)	8 (3–0)
5 min	8 (0–0)	9 (0–)
Perinatal outcome	63	52
Live births	56	49
Still births	0	2
Early neonatal death	1	1

Thrombocytopaenia = < 150 × 10^9^/l.

Among the normotensive group, most indications (8/14) were for foetal distress and cephalopelvic disproportion. Among the pre-eclamptic group severe pre-eclampsia/eclampsia and foetal distress accounted for most (17/19) indications for CS. After delivery, two PE patients were admitted to an intensive care unit and five required high-care nursing.

As expected, the birthweight was significantly lower in the pre-eclamptic pregnancies compared to the normotensives (2.6 ± 0.8 vs 3.14 ± 0.42 kg; *p* < 0.0001). One neonate in the pre-eclamptic group was born with Down syndrome.

There were two neonatal deaths. Patient 1, a 25-year-old P0G1, who was HIV infected presented at 28 weeks of gestation in pulmonary oedema. The blood pressure value was 224/134 mmHg, pulse rate was 106 beats per minute, proteinuria 2+ and oedema 1+. Her tissue Doppler E/E_a_ ratio was 42, RI was 0.74, uric acid level was 0.43 mmol/l and platelet count was 122 × 10^9^/l. Rapid-acting blood pressure-lowering drugs (adalat, hydrallazine, labetalol, aldomet and MgSO_4_) were administerd and she was induced at 30 weeks. She delivered a 1-kg baby with poor Apgar scores who died an hour later.

The second patient, a 24-year-old P0G1 who was HIV uninfected presented at 40 weeks of gestation with a BP of 126/82 mmHg. Her tissue Doppler E/Ea was 15.4, RI was 0.67, and ejection fraction was 68%. She delivered vaginally a 3.4-kg baby with low APGAR scores who died two days later in respiratory distress.

## Discussion

**Fig. 2.**
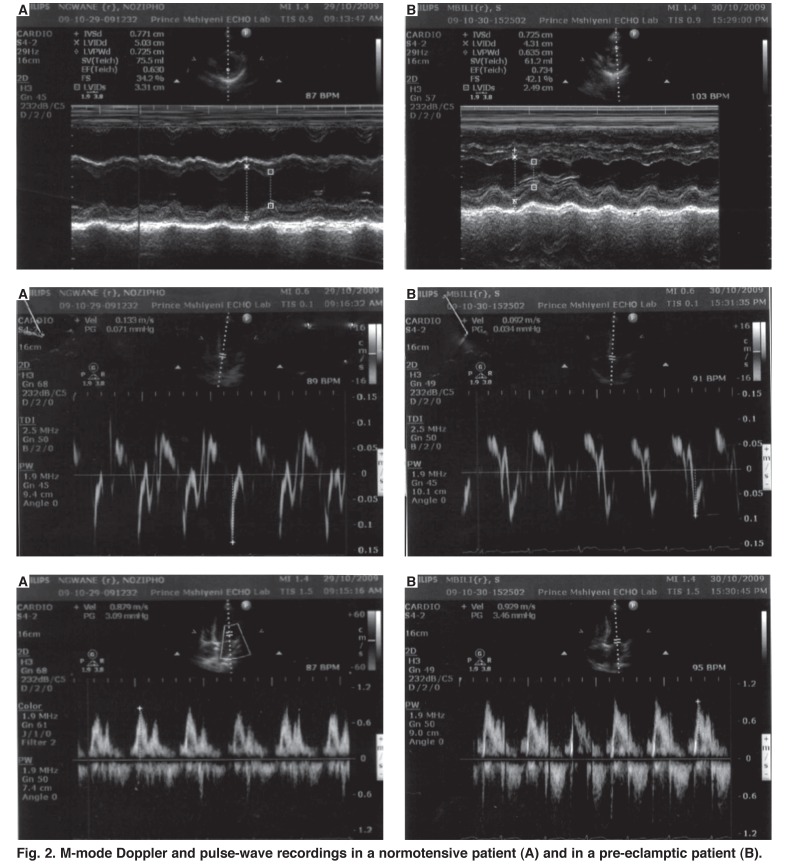
M-mode Doppler and pulse-wave recordings in a normotensive patient (A) and in a pre-eclamptic patient (B).

This was the first study to evaluate haemodynamic alterations in pre-eclampsia using simultaneous BNP and tissue Doppler markers of the left ventricular filling pressure (Fig. 2). The findings indicate that despite the contracted blood volumes in PE, the haemodynamic changes that accompany vasoconstriction in PE are associated with significant changes in LV filling, as reflected by the TDI and BNP levels. The study confirms that the intense vasoconstriction in PE is accompanied by increased left ventricular afterload, accompanied by a reduced cardiac output, hypovolaemia and increased cardiac filling pressures.

Recent studies have confirmed that BNP concentrations are elevated in PE and other hypertensive disorders of pregnancy.^19^-^22^ The increased serum BNP levels in PE are greater than that documented in normotensive pregnancies,^23^,^24^ suggesting that BNP activation in PE is a response to changes in the maternal circulation that reflect the pathophysiological changes in the utero–placental unit. It is known that median BNP levels are low (< 20 pg/ml) and remain stable throughout gestation in normotensive pregnancies.^24^ BNP levels rise in pregnancies complicated by mild PE and are even higher in severe pre-eclamptics, probably reflecting ventricular strain and/or sub-clinical cardiac dysfunction secondary to the increased afterload that is characteristic of PE.^13^,^24^

Itoh *et al*.^13^ reviewed the charts of 15 obstetric patients who presented with acute dyspnoea, and found that seven had PE, with elevated BNP levels. This correlated with acute ventricular overload and they responded well to volume management and diuresis. In two patients, markedly elevated serum BNP levels and significant left ventricular dysfunction was found, which was not apparent by standard clinical evaluation.^13^ In patients with severe pregnancy-induced hypertension, an eight-fold increase in BNP levels has been documented, with a positive correlation between the plasma BNP levels and the mean arterial blood pressure (*r* = 0.62, *p* < 0.001).^18^

Until recently, very little was known about BNP levels during pregnancy. Hameed *et al.*^25^ have shown that although pregnant BNP levels are approximately two-fold higher than their non-pregnant counterparts, they do not significantly fluctuate and probably reflect the physiological increase in cardiac output during pregnancy. Our study also confirms that BNP levels remain normal in the peripartum period in normotensive pregnant women.

Circulating plasma BNP levels are mildly elevated in healthy pregnancies compared to non-pregnant controls. A longitudinal study of 29 healthy pregnant women in each trimester and in the postpartum period has shown no significant differences in the median BNP levels in the various stages of pregnancy and postpartum [first trimester 20 (10–115) pg/ml]. Although pregnant BNP levels were twice as high as the non-pregnant BNP levels, there were no significant differences among the cases compared to non-pregnant controls. These authors concluded that pregnancy is associated with a small increase in the BNP levels compared with non-pregnant women.^25^

By contrast, women with PE show adaptations to the increase in systemic blood pressure with significant modification of left ventricular structure and function.^26^ Echocardiographic studies show statistically significant increases in LV mass, increased LV end-systolic and end-diastolic volumes, accompanied by significant reductions in LV ejection fraction and percentage of fractional shortening.^27^-^29^

We also found an increase in the size of the left atrium in the pre-eclamptic group and although not statistically significant, this probably reflects early cardiac structural changes in PE. More importantly, we showed elevated tissue Doppler E/Ea ratios in the pre-eclamptic patients compared to the normotensive group of patients (11.02 ± 5.6 vs 9.16 ± 2.6, *p* < 0.05). This increase was attributed to a rise in the tissue Doppler E wave and probably reflects rising cardiac filling pressures in PE.

All other differences in the echocardiographic parameters were statistically insignificant, pointing to the sensitivity of tissue Doppler in detecting early changes in diastolic filling pressures. Although tissue Doppler-derived E_a_ correlates with the ventricular time constant and is a relatively load-independent measure of myocardial relaxation in patients with cardiac disease, in our study it did not appear as sensitive as BNP, which is emerging as a new marker in identifying early haemodynamic changes. In patients with PE, BNP has been observed to be linearly related to the left ventricular structural and functional changes observed.^16^

In this study we have shown that plasma BNP levels were significantly increased in pre-eclamptic compared to normotensive patients in the antepartum period, and decreased significantly in the postpartum period. Women with PE have increased sensitivity to angiotensin II, resulting in increased peripheral vasoconstriction and volume retention.^30^,^31^

BNP is known to suppress renin release and one reason for its activation could be the derangement in the renin angiotensin system that occurs in PE.^31^ To what extent the pro-inflammatory cytokines and endothein-1 are known to stimulate natriuretic peptide release is not clear, nor is their relationship with cardiac filling pressures known.

While it appears that the increase in circulating plasma BNP is most likely explained by the rising cardiac filling pressure, it should be remembered that PE is documented to be a relatively volume-contracted state with marked peripheral vasoconstriction. In this regard, elevation in BNP levels may be due to myocardial remodelling and sub-clinical ventricular dysfunction that accompanies the severe vasoconstriction that is observed in PE.

Our findings suggest that BNP reflects the myocardial load in PE. This hypothesis is confirmed by the decrease in BNP levels in the puerperium when the placenta has been removed.

As expected, significant changes were seen in the pre-eclamptic group with regard to blood pressure, pedal oedema, proteinuria, uric acid and serum creatinine levels, highlighting the need for intervention, as these factors are documented indicators of worsening disease progression in PE. The relationship between proteinuria and the heart need further evaluation, as well as its relationship to HIV infection. A significant number of our patients were HIV infected but there were no significant changes in the BNP or TDI levels, or even in outcome in these subjects.

In our study, seven pre-eclamptic patients required emergency care post delivery, in keeping with the association between PE and adverse pregnancy events. Of the seven pre-eclamptics who required emergency care, two required ICU admission. An increased rate of Caesarean sections, lower Apgar scores, lower birth weight and an increased admission rate to neonatal ICU has been described in PE.33 Numerous studies have documented similar pregnancy sequelae in patients with PE.^32^, ^33^

Recently, foetal wellbeing by weekly assessment of umbilical artery Doppler examinations and biophysical profiles were investigated by Sezik *et al.* in patients with pre-eclampsia in their third trimester.^33^ It was found that 38% of patients had an abnormal Doppler on the last evaluation before delivery. In this group, significantly higher blood pressures and serum uric acid levels were recorded, lower platelet counts, higher incidence of IUGR, lower Apgar scores at five minutes, a higher incidence of perinatal deaths and higher operative delivery rates.^33^

We also found umbilical artery velocity to be more practical in evaluating the foetus in late pregnancy than uterine artery Doppler studies, which have been used to predict PE and IUGR.^30^-^33^ Technical difficulties in obtaining access to uterine Doppler flow readings precluded the use of this measurement in our study. The increase in the resistance index on umbilical Doppler velocimetry is well described,^32^,^33^ and abnormal Doppler umbilical artery waveforms are a strong predictor of perinatal mortality and are associated with a poor perinatal outome.^32^

Previous studies have shown that BNP levels were elevated in patients with pre-eclampsia but the numbers have been few and they have not correlated these changes with TDI.^15^,^17^,^24^

## Limitations of the study

BNP is significantly affected by age, gender, renal function and obesity. Given its correlation with multiple cardiac variables, BNP has high sensitivity but low specificity for the detection of elevated left ventricular filling pressures. In our study this variability was avoided by selecting primiparous patients who were of similar age and constituted a fairly homogenous group of black patients, permitting comparisons to be made.

Also, as mentioned above, most of the changes in BNP that occurred were within normal ranges for our laboratory. It is likely that BNP activation would be more pronounced in severe PE. Furthermore, this study was conducted on patients in the third trimester. Recruitment earlier in pregnancy might have shown whether or not BNP could be used as a marker of PE.

## Conclusion

This study showed that there were significant differences in BNP levels during PE and this probably reflects the haemodynamic changes in this condition. Serum BNP levels were significantly elevated in pregnancies complicated by PE, particularly in those with more severe disease. This increase was reflected in elevated tissue Doppler estimations of LV filling pressure (E/E_a_ ratio).
